# Different virulence levels of *Enterococcus cecorum* strains in experimentally infected meat-type chickens

**DOI:** 10.1371/journal.pone.0259904

**Published:** 2021-11-12

**Authors:** Jana Schreier, Silke Rautenschlein, Arne Jung

**Affiliations:** Clinic for Poultry, University of Veterinary Medicine Hannover, Foundation, Hannover, Germany; USDA-Agricultural Research Service, UNITED STATES

## Abstract

In recent years, pathogenic strains of *Enterococcus cecorum* (EC) have emerged as a causing agent of septicemia and skeletal infection in broiler chickens with a high economic impact worldwide. Although research has been conducted, many aspects of the pathogenesis of the EC-associated disease are still unknown. In the present study, an experimental infection model was established in broiler chickens. Two different EC strains (EC14 and EC15) were compared in two different concentrations of each strain (2 × 10^6^ and 2 × 10^8^ colony-forming units per milliliter (CFU/mL)) after oral infection of one-day-old chicks. Clinical signs and gross lesions of the EC-associated disease were monitored in the following seven weeks. Although both EC strains were originally isolated from clinical disease outbreaks and had a high embryonic lethality, only EC14 successfully induced the typical course of the EC-associated disease with characteristic clinical signs and gross lesions. In total, 23% of the birds in the two EC14-groups were EC-positive in extraintestinal organs on culture, and no differences were found between the two infectious doses. EC14 was frequently detected via real-time PCR in the free thoracic vertebra (FTV) and femoral heads without any detectable gross lesions. The number of EC positive spleens from infected broilers was comparable using bacterial isolation and a specific real-time PCR. Interestingly, EC15 was not detected in extraintestinal organs, although birds in the EC15 groups were colonized by EC in the ceca after experimental infection. The present study represents first proof that virulence differs among EC strains in experimentally infected chickens, and emphasizes the need to further characterize virulence factors and pathogenic mechanisms of EC. The strain EC14 at a dose of 10^6^ CFU is suitable for reproduction of the EC-associated disease. The experimental infection model reported here provides the basis for further research on the EC pathogenesis and possible prevention and intervention strategies.

## Introduction

Once considered a gut commensal in chickens, *Enterococcus cecorum* (EC) has turned out to be a serious threat to the broiler industry in the past two decades [1–4]. To date, outbreaks of the EC-associated skeletal disease have been reported in broilers and broiler breeder chickens worldwide, and are commonly referred to as one of the main infectious causes of economic losses in the industry [5–9]. After a septic phase in the first to third week of the production cycle, EC causes osteomyelitic lesions at several predisposed articulations in the spine and the legs of meat-type chickens, including the joints of the free thoracic vertebra (FTV), the hip joints and the knee joints. During septicemia, EC can be isolated from the liver, heart or spleen, where it is the cause for hepatitis, pericarditis, and splenomegaly [9, 10]. Clinical symptoms at this stage are non-specific. Birds can be asymptomatic or depressed, with ruffled feathers and closed eyes [11]. During the skeletal phase, affected birds are lame or completely paralyzed and often found in a typical sitting position on their hocks with both legs extended to the front [12]. EC can be isolated from inflammatory lesions at the FTV, the femoral heads or the tibial heads [13]. Different EC-detection methods have been used successfully in previous studies, including bacterial cultivation and biochemical detection methods [4, 7, 14, 15], 16S rRNA partial gene sequencing [9], and quantitative real-time PCR [16]. Whenever EC is detected as the causing agent of a disease outbreak, early treatment is crucial to reduce the associated mortality and therapy costs. Therapy of severely affected birds is not promising. As different antimicrobial resistance profiles have been reported, antimicrobial susceptibility testing of the respective EC strain is highly recommended for the correct choice of treatment [17].

To date, little is known about the EC pathogenesis. In broilers, transmission via the fecal-oral route is most likely, although infection via the respiratory tract has also been discussed [3, 9]. Potential predisposing factors for the development of the EC-associated disease, such as environmental conditions, co-infection with other pathogens, and osteochondrotic lesions at the FTV, have been discussed and investigated [10, 18, 19]. However, it remains unclear which internal and external factors enable EC to translocate from the gut to other tissues [18]. In the literature, usually a distinction is made between pathogenic and commensal strains, but there is limited information on specific characteristics (e.g., virulence factors, antimicrobial resistance or serotypes) [20–22]. Pathogenic EC strains preferentially colonize the gut in the first week of life, whereas commensal strains tend to colonize from the third week onwards. Pathogenic strains are thought to be highly effective in colonizing the gut and spreading within the flock [10]. In order to determine the virulence of an EC-strain, scientists often focus on clinical symptoms and gross lesions caused by EC. Strains are usually considered pathogenic when they are recovered from extraintestinal organs, whereas commensal strains are generally isolated from the intestines. Pulsed-field gel electrophoresis (PFGE) has shown that strains recovered from extraintestinal organs are genetically diverse from intestinal strains [23–25]. Embryo lethality assays have also been used to characterize the virulence of EC strains. Extraintestinal EC isolates seem to cause higher embryo lethality rates and are therefore thought to be more virulent than intestinal strains [21, 26–28]. Nonetheless, until now, there is no experimental evidence that some strains are more virulent than others in broiler chicks in the first weeks of life.

The first aim of this study was to establish a reliable infection model in meat-type chickens. Therefore, we compared two different EC strains with regard to their pathogenicity. Two different infectious doses of each strain were used to find a suitable dose for further experiments. As a second aim we wanted to evaluate different diagnostic methods with the experimental infection. Bacteriological examination and real-time PCR were compared to detect EC in the spleens of EC-infected birds. Furthermore, our aim was to examine the correlation of macroscopic lesions at the osseous predilection sites and the detection of EC in these tissues.

## Materials and methods

### Animals and housing

Day-old broiler chicks (Ross 308) were obtained from a commercial hatchery (Brüterei Weser-Ems GmbH & Co. KG, Visbek, Germany), randomly divided into five groups and housed in floor pens in five separate isolation units at the Clinic for Poultry, University of Veterinary Medicine Hannover, Foundation, Hannover, Germany. The birds were raised under standard temperature conditions on wood shavings and fed a broiler standard diet (Deuka, Deutsche Tiernahrung Cremer GmbH & Co. KG, Duesseldorf, Germany). Feed and water were provided *ad libitum*. On the day of placement, birds had 24 hours of light. Afterwards, the light program was set to 15 hours of light from 07:30 to 22:30 and a nine-hour dark period throughout the whole trial. Infrared lamps were set up in the pens for the first two days and removed on the third day. The study design was approved by the Animal Ethics Committee of the University of Veterinary Medicine, Foundation, Hannover and conducted in line with regulations on animal welfare and animal experimentation in Germany (33.19-42502-04-19/3170).

### Experimental set-up

In this experiment, 741 one-day-old broiler chicks were used. On the day of arrival, 10 chicks were euthanized to determine their EC-negative status. Yolk sacs were removed for bacteriological examination via culture and detection of EC-DNA via real-time PCR (see respective sections). The remaining 731 one-day-old chicks were randomly divided into five groups consisting of 147 (four infected groups) or 143 animals (control group), respectively. Based on the challenge isolate and the bacterial concentration of the inoculum, the five groups were named as follows: EC14_low (EC14; 2 × 10^6^ colony-forming units per milliliter (CFU/mL)), EC14_high (EC14; 2 × 10^8^ CFU/mL), EC15_low (EC15; 2 × 10^6^ CFU/mL), EC15_high (EC15; 2 × 10^8^ CFU/mL), and control (physiological saline). Each bird received 0.5 mL of the respective inoculum directly in the crop. In the following weeks, the birds were monitored daily for clinical signs of the EC-associated disease. Those animals showing severe signs of apathy or lameness resulting in inappetence were euthanized, and necropsy was performed. Regular necropsies of 20 birds per group were performed weekly at days 11, 18, 25, 32, 39, 46, and 53 post hatch. At necropsy, body weight (BW) was determined and pathologic lesions were documented. Amies medium swabs (Hain Lifesciences GmbH, Nehren, Germany) and tissue samples from the heart, liver, and spleen were taken from all animals throughout the whole trial for bacteriological examination via culture and real-time PCR, respectively. Furthermore, dry swabs (Applimed SA, Châtel-St-Denis, Switzerland) from the cecum were taken from all the broilers. At study days 25, 32, 39, 46, and 53, a scoring of macroscopic bone lesions was performed. The FTV and the femoral heads were exposed and cut sagittally to examine the cartilage and the underlying bone. Dry swabs from the FTV and the femoral heads were taken for real-time PCR analysis. The tip of all dry swabs was cut off and placed in 1.5 mL Eppendorf tubes (Sarstedt AG & Co. KG, Nuembrecht, Germany). Tissue samples and dry swabs were stored at—20°C until DNA extraction.

### Challenge isolates and preparation of the inoculate

For oral inoculation, two different EC strains were used (EC14/086/4/A and EC15/827/1/A). Both strains were isolated from a broiler’s heart that had been examined in the scope of two separate disease outbreaks in 2014 (EC14) and 2015 (EC15), respectively. The affected birds in both disease outbreaks had shown non-specific symptoms and lameness. For EC14 pericarditis, hepatitis, femoral head necrosis and spondylitis at the FTV had been found at necropsy in several birds throughout the production cycle [9]. EC15 had been isolated from the heart of a 14 days old broiler. The two strains showed a comparable high embryo lethality (EC14 = 86.7%, EC15 = 80.0%, respectively), as previously published [21]. After isolation, both strains were confirmed as EC using biochemical methods (Rapid ID32 Strep®; BioMérieux GmbH, Nuertingen, Germany) and 16S rRNA partial gene sequencing. Sequences are available at GenBank under accession numbers KX674314 and KX674323 via the following links: https://www.ncbi.nlm.nih.gov/nuccore/KX674314 and https://www.ncbi.nlm.nih.gov/nuccore/KX674323 [21]. EC strains were stored at– 80°C using the CryoBank® system (Mast Diagnostica GmbH, Reinfeld, Germany). Two days prior to inoculation, the bacterial strains were inoculated on Columbia sheep blood agar (Oxoid GmbH, Wesel, Germany). The plates were incubated at 37°C for 20 hours under microaerophilic conditions in a desiccator using the candle method. On the following day, subcultures were made, to be used for the inoculum preparation. On the day of arrival of the chickens, four different bacterial solutions were prepared using sterile physiological saline at room temperature. Colony material was dissolved in physiological saline to set an optical density of 1.1 McFarland (McF; DENSIMAT; BioMérieux GmbH, Nuertingen, Germany). This optical density was determined in preliminary tests and corresponds to an EC concentration of approximately 2 × 10^8^ CFU/mL. For each strain, a second bacterial solution containing 2 × 10^6^ CFU/mL was prepared by diluting the initial solutions 1:100. A total of 150 mL of each solution was filled in three 50 mL falcon tubes (Nerbe plus GmbH & Co. KG, Winsen, Germany) and stored at room temperature until infecting the birds. The actual concentrations of the four solutions were confirmed by determining the total bacterial count. A 10-fold dilution series up to 10^−10^ of the inoculum was produced and 100 μL of each dilution was plated on two Columbia sheep blood agar plates. Colonies were counted after incubation at 37°C for 24 hours under microaerophilic conditions, and the concentration of each bacterial solution was calculated in CFU/mL.

### Qualitative microbiology via culture

For EC-reisolation, Amies medium swabs from different organs (heart, liver and spleen) were inoculated onto Columbia colistin-nalidixic acid (CNA) agar (Oxoid GmbH, Wesel, Germany). After incubation at 37°C for 24 hours under microaerophilic conditions, plates were screened for colonies showing a morphology typical for EC. Subcultures were produced on Columbia sheep blood agar and incubated for another 24 hours. From pure subcultures, catalase and oxidase testing as well as Gram staining was performed. Isolates were considered EC when they fulfilled the following criteria: small, gray, mucoid colonies with slight alpha-hemolysis; catalase and oxidase negative; gram positive to gram labile ovoid cocci. Isolates giving questionable results were further analyzed via 16S rRNA partial gene sequencing at Microsynth AG, Lindau, Germany [21, 29–31].

### DNA isolation and quantitative real-time PCR

DNA was isolated from tissue samples (spleen) and dry swabs (cecum, FTV, femoral head) taken at necropsy. A commercial isolation kit (InnuPrep DNA Mini Kit, Analytik Jena AG, Jena, Germany) was used in accordance with the manufacturer’s instructions, with some modifications. For tissue samples, the first step was to cut off a piece the size of a rice grain and place it in tubes containing Precellys Bulk Beads (1.4 mm, Bertin Technologies SAS, Montigny-le Bretonneux, France) for tissue homogenization. Lysis buffer (400 μL volume) was added and tissue homogenization was performed with the Precellys 24 tissue homogenizer (Bertin Technologies SAS) using the following conditions: 5500 rpm (rounds per minute), three sessions of 20 seconds each with five second breaks between the sessions. Homogenization was followed by centrifugation at 4°C for 15 minutes at 13800 × g to reverse the foaming. The supernatant was transferred into an Eppendorf tube and 25 μL Proteinase K (20 mg/mL) were added. Lysis was performed at 50°C on an Eppendorf Thermomixer compact (Eppendorf AG, Hamburg, Germany) at 500 rpm for 1 h. For swabs, the first step was to cut half of the tip and place it in an Eppendorf tube. A total of 400 μL of lysis buffer and 25 μL Proteinase K (20 mg/mL) were added. Lysis was performed at 50°C at 500 rpm for 10 minutes. All subsequent steps were identical for tissue samples and swab samples. A total of 400 μL of binding solution was added to the lysate; thereafter the solution was vortexed for 15 seconds. The solution was added to the spin filter and centrifuged at 11000 × g for two minutes. Two washing steps were performed with the two different washing buffers. A total of 500 μL of washing solution HS and 750 μL of washing solution MS were added to the spin filter, followed by centrifugation at 11000 × g for one minute. In the final step, 30 μL of the elution buffer was added to the spin filter. After a one-minute incubation period, the samples were centrifuged at 6000 × g for one minute. The total amount of DNA was determined using NanoDrop® ND-1000 Spectrophotometer (Thermo Fisher Scientific Inc., Wilmington, NC, USA) and DNA was stored at -20°C until further use.

Real-time PCR was performed for each sample in duplicate on 96-well-plates (Applied Biosystems™, Fisher Scientific GmbH, Schwerte, Germany) using the QuantStudio 3 Real-Time-PCR-System (Thermo Fisher Scientific Inc.). Briefly, the same primers, probes and polymerase were used as previously described [16]. Each well contained the following mixture of reagents: 0.25 μL EGFP-1-F, 0.25 μL EGFP-10-R, 0.25 μL EGFP-Hex (primers and probe for internal control), 0.5 μL qEcec_for, 0.5 μL qEcec_rev, 0.25 μL qEcec_probe (EC specific primers and probe), 5 μL PerfeCTa ToughMix (Quanta Biosciences Inc., Beverly, MA, USA), 0.25 μL internal control Intype IC-DNA (Qiagen GmbH, Hilden, Germany) and 1 μL template in a total volume of 10 μL. The cycling conditions were as follows: initial denaturation at 95°C for 10 minutes, followed by 40 cycles of 95°C for 15 s, and 60°C for 60 s. Mean Ct values above 36 were considered negative.

### Statistical analysis

Statistical analysis of data was performed using SAS Enterprise Guide (Version 7.15, SAS Institute Inc., Cary, NC, USA) and graphs were created with GraphPad Prism (Version 9.2, GraphPad Software, LLC, San Diego, CA, USA). Descriptive statistics were used to analyze clinical signs and gross lesions. For evaluation of significant differences in BW between groups and between EC-positive and EC-negative birds in the EC14 groups, we performed the Kruskal-Wallis test and Mann-Whitney *U* test, as conditions of normality and heterogeneity of variance were not met. Isolation rates of culture and EC-DNA detection in real-time PCR were compared between groups using the Fisher’s Exact Test. The Bonferroni-Holm correction method was used to adjust *p*-values for multiple testing where applicable [32]. Results from bacteriological examination and real-time PCR of the spleen within the same group were compared using the Kappa coefficient and McNemar’s Test. The Kappa coefficient was interpreted as published by Landis and Koch in 1977 [33]. Differences were considered significant at *p* ≤ 0.05.

## Results

### Clinical signs of the EC-associated disease

**Fig 1:**
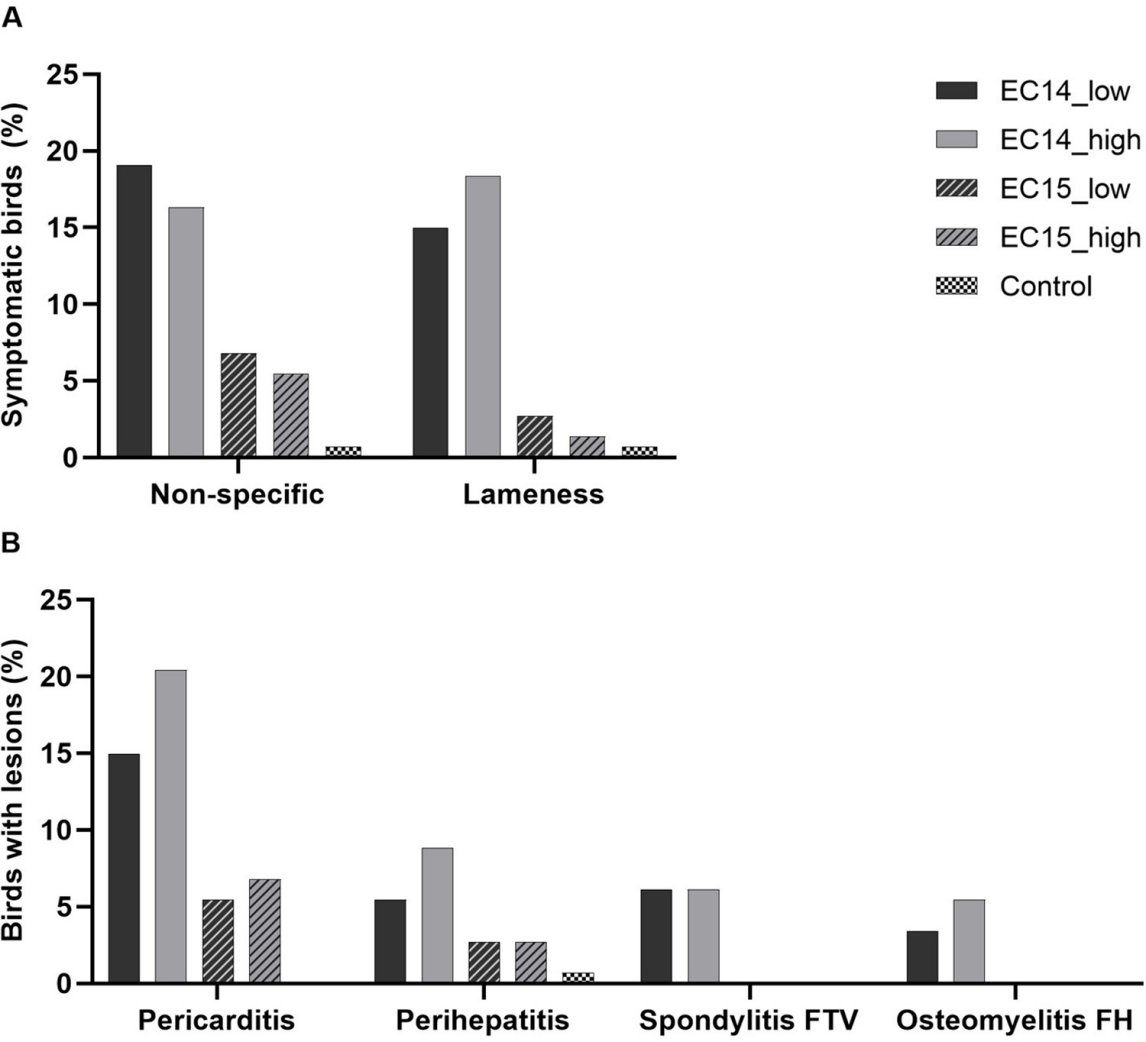
Clinical signs (A) and pathologic lesions (B) observed during the experiment. (A) Non-specific symptoms include depression, ruffled feathers, and closed eyes. Lameness includes all stages from mild to completely paralyzed. (B) Spondylitis FTV = abscess was found at the free thoracic vertebra, FH = femoral head. N = 147 per group in the EC-infected groups, N = 143 in the control group.

**Fig 2:**
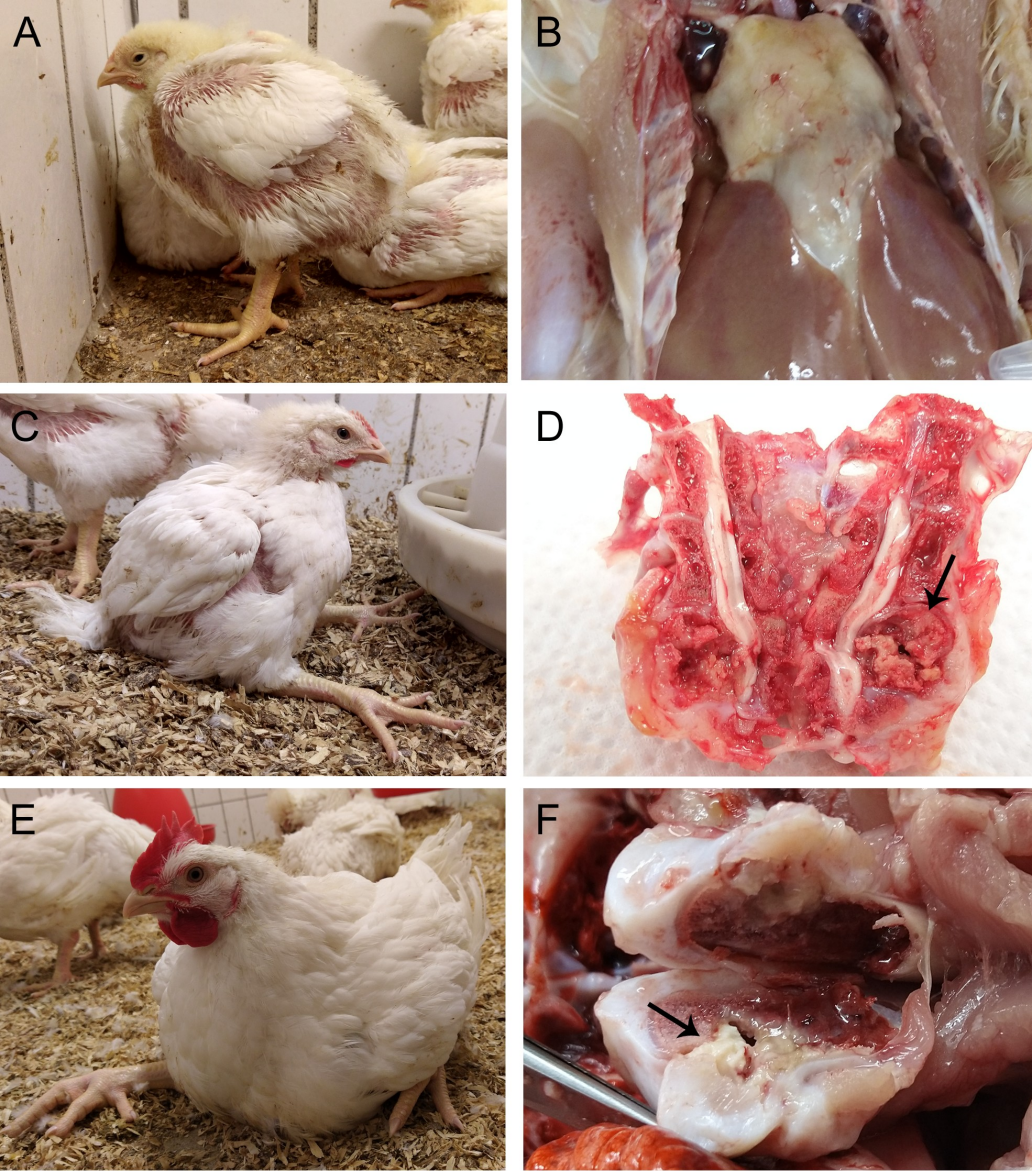
Clinical signs and pathology. (A) Clinical signs of the septic phase of the EC-infection (study day 15, EC14_low). (B) Pericarditis due to EC (study day 25, EC14_low). (C) Clinical signs of the skeletal phase of the EC-infection. This broiler was sitting on hocks and unable to walk (study day 29, EC14_low). (D) Spondylitis at the FTV (arrow). The vertebral column was removed and split sagittally to expose the lesion (study day 30, EC14_high). (E) Clinical signs of the skeletal phase of the EC-infection. This broiler was lame due to femoral head osteomyelitis (study day 52, EC14_high). (F) Femoral head osteomyelitis (arrow). The femoral head was exarticulated by using a sterile scalpel and split sagittally (study day 43, EC14_low). EC was isolated in pure culture from the lesions in pictures B, D, and F.

Birds were monitored daily throughout the trial for non-specific symptoms of septicemia (depression with ruffled feathers and closed eyes) and specific symptoms of the skeletal phase (progressive lameness up to paralysis) of the EC-infection. Non-specific symptoms as well as lameness were observed in all five groups, but the amount of birds showing these symptoms was higher in the EC14 groups (Figs 1A, 2A, 2C, and 2E). Severely affected birds were euthanized for animal welfare reasons. Additionally, some birds died during the trial in all five groups. Necropsy was performed on all these birds to determine the cause of death and samples were taken according to the protocol at regular necropsies. The total number of dead and euthanized animals in each group was slightly higher in the EC14 groups compared to the EC15 groups and the control group (8/147 in EC14_low, 11/147 in EC14_high, 4/147 in EC15_low, 6/147 in EC15_high, and 4/143 in the control group). Although there were birds that had to be euthanized or that died during the experiment in all five groups, the EC-associated disease was only detected in birds from the two EC14 groups (6/8 (75%) in EC14_low, and 7/11 (64%) in EC14_high, respectively). Table 1 gives an overview of the day of death, the clinical signs, and the pathologic lesions found in these affected animals. Chickens in the EC15 groups and the control group generally had to be euthanized or died because of other reasons than EC. The main cause of death in these groups was the ascites syndrome.

**Table 1:** EC-positive euthanized or dead birds at irregular necropsies.

Consecutive animal number	Study day	Group	Clinical signs	Morphological diagnosis during necropsy	EC-positive organs (culture)^a^
1	3	EC14_high	Depression	Omphalitis/yolk sac infection, ascites	Yolk sac
2	15	EC14_low	Depression, lameness	Splenomegaly	Heart, Liver, Spleen
3	22	EC14_high	Depression, respiratory distress	Marbled liver and spleen	Heart, Spleen
4	30	EC14_high	None	Pericarditis, perihepatitis, ascites	Heart, Liver, Spleen
5	30	EC14_high	Depression, progressive lameness up to paralysis	Pericarditis, spondylitis	FTV
6	36	EC14_low	Depression, progressive lameness up to paralysis	Pericarditis, spondylitis	Spleen, FTV
7	36	EC14_low	Depression, progressive lameness up to paralysis	Pericarditis, spondylitis	Liver, spleen, FTV
8	36	EC14_high	Depression, progressive lameness up to paralysis	Pericarditis, perihepatitis, spondylitis	Spleen, FTV
9	36	EC14_high	Depression, progressive lameness up to paralysis	Pericarditis, spondylitis, femoral head osteomyelitis	Spleen, FTV
10	37	EC14_low	Depression, progressive lameness up to paralysis	Pericarditis, spondylitis, femoral head osteomyelitis	Spleen, FTV
11	43	EC14_high	Depression, progressive lameness up to paralysis	Spondylitis	FTV
12	43	EC14_low	Depression, progressive lameness up to paralysis	Pericarditis, spondylitis	FTV
13	43	EC14_low	Depression, progressive lameness	Pericarditis, femoral head osteomyelitis	Left femoral head

This table summarizes the study day of irregular necropsy, group assignment, clinical signs, gross lesions, and EC-positive organs found in bacteriological examination via culture for all EC-positive birds that were found dead or had to be euthanized due to animal welfare reasons in the EC14-infected groups. Heart, liver and spleen from all 13 birds were bacteriologically examined via culture. The free thoracic vertebra (FTV) and the femoral heads were only sampled and bacteriologically examined via culture when gross lesions (spondylitis, femoral head osteomyelitis) were detected.

^a^All sampled organs that are not named in this column were EC-negative on culture.

### Gross lesions

At necropsy, we found serous pericarditis and fibrinous perihepatitis mainly during the septic phase from the second to the fourth week (Fig 2B). The predominant gross lesion in groups EC14_low and EC14_high was pericarditis (15.0% and 20.4%, respectively; Fig 1B). Perihepatitis was seen less often in the two EC14_groups (5.4% and 8.8%, respectively). Pericarditis and perihepatitis were also found in the EC15 groups, but to a lesser extent (Fig 1B). During the skeletal phase (week four to seven), spondylitis and femoral head osteomyelitis could be detected macroscopically in some of the birds in the EC14 groups (Fig 2D and 2F). Spinal abscesses were found in 6.1% of the birds in both EC14 groups. Femoral head osteomyelitis was detected even less frequently (3.4% in group EC14_low and 5.4% in group EC14_high, respectively). No bone lesions were detected in the EC15 groups and the control group (Fig 1B). At study day 53, the birds reached a final BW of 4.1 ± 0.5 kg in group EC14_low, 4.2 ± 0.7 kg in group EC14_high, and 4.6 ± 0.6 kg in the control group, respectively. Concerning BW, no significant differences between the EC14 groups and the control group were seen for all necropsy days. When comparing EC-positive and EC-negative birds in the two EC14 groups, significantly lower BW were found in the EC-positive birds at study days 18,32, 39, and 46 (S1 Fig). No significant differences were found for the other three necropsy days (11, 25, and 53). As the strain EC15 did not cause any typical clinical signs or gross lesions associated with EC, we decided to exclude EC15 from all further analyses.

### Qualitative microbiology via culture

**Fig 3:**
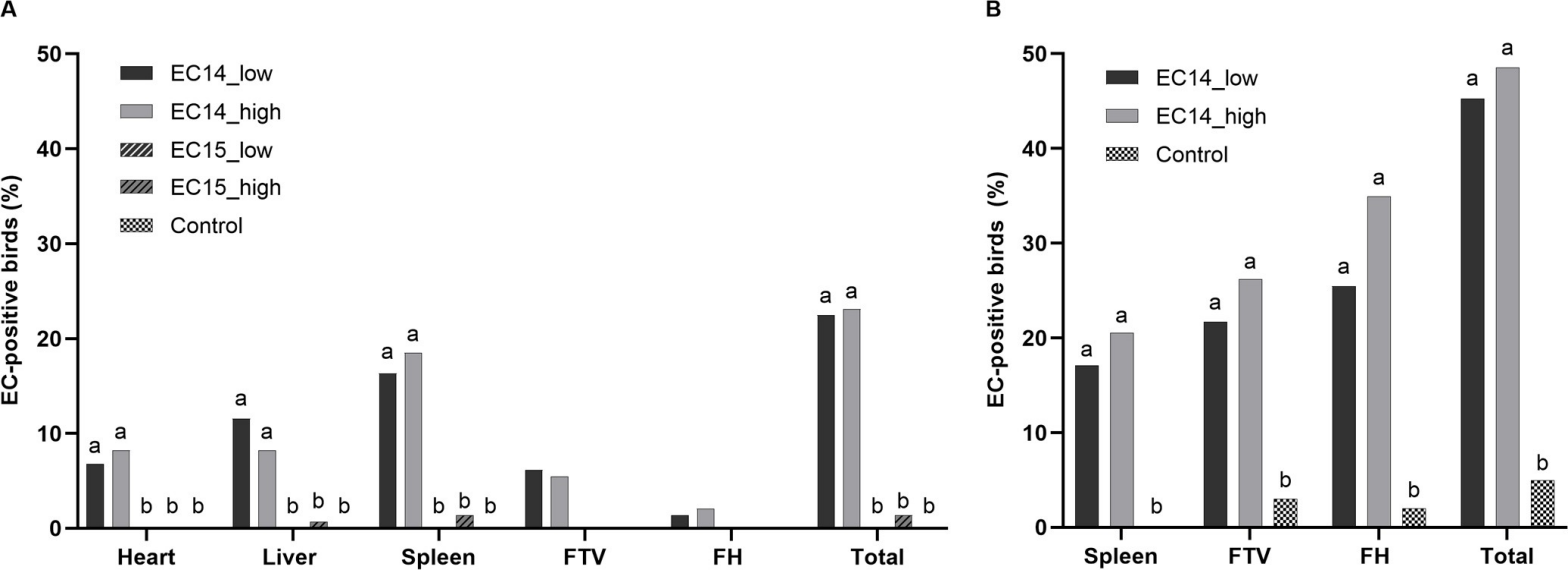
Bacteriological examination via culture and real-time PCR. (A) EC-positive birds on culture in %. (B) EC-positive birds in real-time PCR in %. Ct values below 36 were considered positive. Different letters indicate significant differences between the groups per organ (*p* ≤ 0.05). Comparison between the groups was made for each organ by using Fisher’s exact test. *p*-value adjustments for multiple testing were performed by using the Bonferroni-Holm correction method. Comparison between the groups was not done for culture results of the FTV and the femoral heads, because these samples were only taken when gross lesions were detected. N = 147 per group (EC-infected groups), N = 143 in the control group. FTV = free thoracic vertebra, FH = femoral heads.

**Fig 4:**
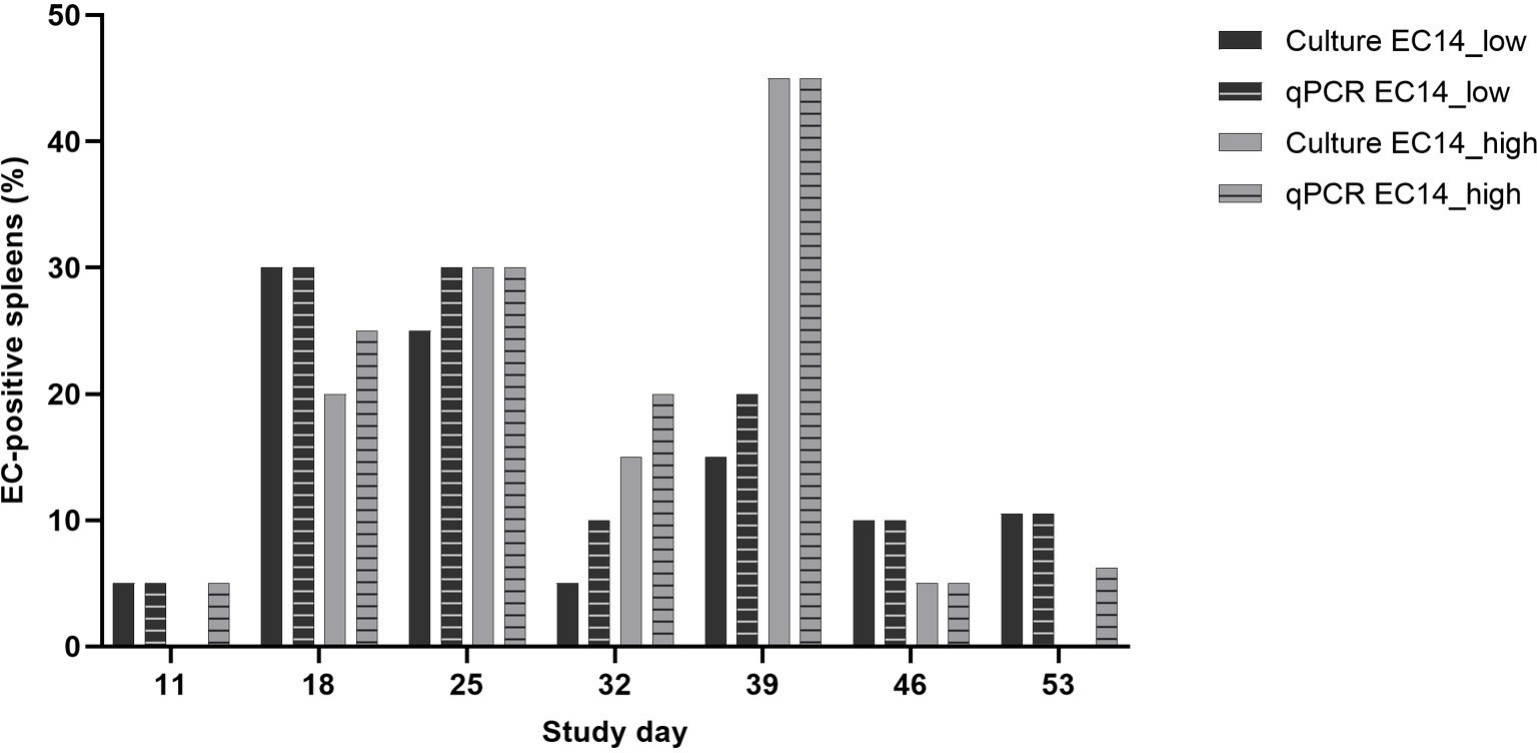
Comparison of bacteriological examination of the spleen via culture and real-time PCR. EC-positive spleens in groups EC14_low and EC14_high are shown per study day in %. Ct values below 36 were considered positive. All birds in the control group were found to be EC-negative in both detection methods. According to the Kappa coefficient, there was at least a substantial agreement (κ > 0.61) on the EC-status at all study days and McNemar’s test revealed no significant differences in distribution of results. N = 20 per group and study day.

The heart, liver, and spleen from all the birds were sampled at all necropsies throughout the trial. The FTV and the femoral heads were sampled only when gross lesions were found. In total, approximately 23% of the broilers in the EC14 groups were positive for EC on culture (22.5% in group EC14_low and 23.1% in group EC14_high, respectively; Fig 3A). EC was mainly isolated from the spleen (16.3% in group EC14_low and 18.5% in group EC14_high, respectively), followed by the liver (11.6% in group EC14_low and 8.2% in group EC14_high, respectively) and the heart (6.8% in group EC14_low and 8.2% in group EC14_high, respectively). Although pericarditis was seen in more than 15% of the birds in the EC14 groups, EC was isolated from less than 10% of the hearts in both groups. In contrast, more than 10% of the livers were EC-positive, whereas perihepatitis was seen in less than 10% of the livers. EC was not recovered from any of the organs in the EC15_low group or in the control group. In the EC15_high group, EC was recovered from two of 147 birds (1.4%). Regarding the whole trial, the EC14 groups had significantly higher isolation rates from the heart, liver, and spleen compared to the EC15 groups and the control group (*p* ≤ 0.05). There was no difference between the two EC14 groups for the heart, liver and spleen (Fig 3A). In the course of the experiment, EC was recovered at all necropsies from some of the birds in the EC14 groups. The isolation rate peaked at study days 25 and 39 (Fig 4). At study day 39, 50% (10/20) of the birds in group EC14_high and 35% (7/20) of the birds in group EC14_low were EC-positive on culture. At this time point, EC was mainly recovered from the spleen, but there were also some birds that already had developed EC-positive gross lesions at the FTV. In total, 6.1% of the birds in group EC14_low and 5.4% of the birds in group EC14_high had EC-positive lesions at the FTV. EC-associated femoral head osteomyelitis was found less frequently in 1.4% (EC14_low) and 2.1% (EC14_high) of the birds, respectively.

### Real-time PCR: Spleen, FTV, and femoral heads

In total, approximately 19% of the spleens, 24% of the FTVs, and 30% of the femoral heads were EC-positive in the EC14 groups (study days 25–53; Fig 3B). On average, 47% of the birds in the EC14 groups were EC-positive in one or more of the extraintestinal organs examined in real-time PCR. This overall detection rate via real-time PCR was twice as high as the overall detection rate via culture (approximately 23%). The percentage of EC-positive birds in the EC14 groups was significantly higher compared to the control group in all examined organs (*p* ≤ 0.05). The number of EC-positive spleens, FTVs, and femoral heads was slightly higher in the EC14_high group, but no significant difference was found between the two EC14 groups. The number of EC-positive spleens peaked at study day 18 (EC14_low) and at study day 39 (EC14_high; Fig 4). Whereas the number of EC-positive FTVs peaked at study day 32 (EC14_high), the peak in group EC14_low was seen at study day 46. The number of EC-positive femoral heads peaked at study day 39 in the EC14_low group. In group EC14_high, the highest number of EC-positive femoral heads was found at study day 53 (S2 Fig). EC detection rates from spleen samples via culture and real-time PCR were comparable within each EC14 group at all the study days (Table 2). In real-time PCR, we could find significantly more EC-positive FTVs and femoral heads than were macroscopically detectable (Table 2, S2 Fig).

**Table 2:** Kappa coefficient and McNemar’s test for comparison of different detection methods.

Sampling Site	EC14_low			EC14_high		
	Kappa	McNemar	Comment	Kappa	McNemar	Comment
Spleen	0.8505	0.4142	Perfect agreement on EC-status, no significant differences in distribution of results.	0.6297	0.4669	Substantial agreement on EC-status, no significant differences in distribution of results.
FTV	0.5017	0.0002	Moderate agreement on EC-status, significantly more EC-positive FTVs detected via real-time PCR than gross pathology.	0.3832	< 0.0001	Fair agreement, significantly more EC-positive FTVs detected via real-time PCR than gross pathology.
FH	0.1062	<0.0001	Slight agreement, significantly more EC-positive FHs detected via real-time PCR than gross pathology.	0.1058	< 0.0001	Slight agreement, significantly more EC-positive FHs detected via real-time PCR than gross pathology.

Results from the culture and real-time PCR were compared for the spleens. For FTV and FH, EC-associated gross lesions were compared with the real-time PCR results. The kappa coefficient was interpreted according to Landis and Koch 1977 [33]. FTV = free thoracic vertebra, FH = femoral heads. EC14_low: N = 146 for spleens, N = 106 for FTV and FH; EC14_high: N = 146 for spleens, N = 103 for FTV and FH. FTV and FH were only sampled at study days 25–53.

### Real-time PCR: Cecal colonization

**Fig 5:**
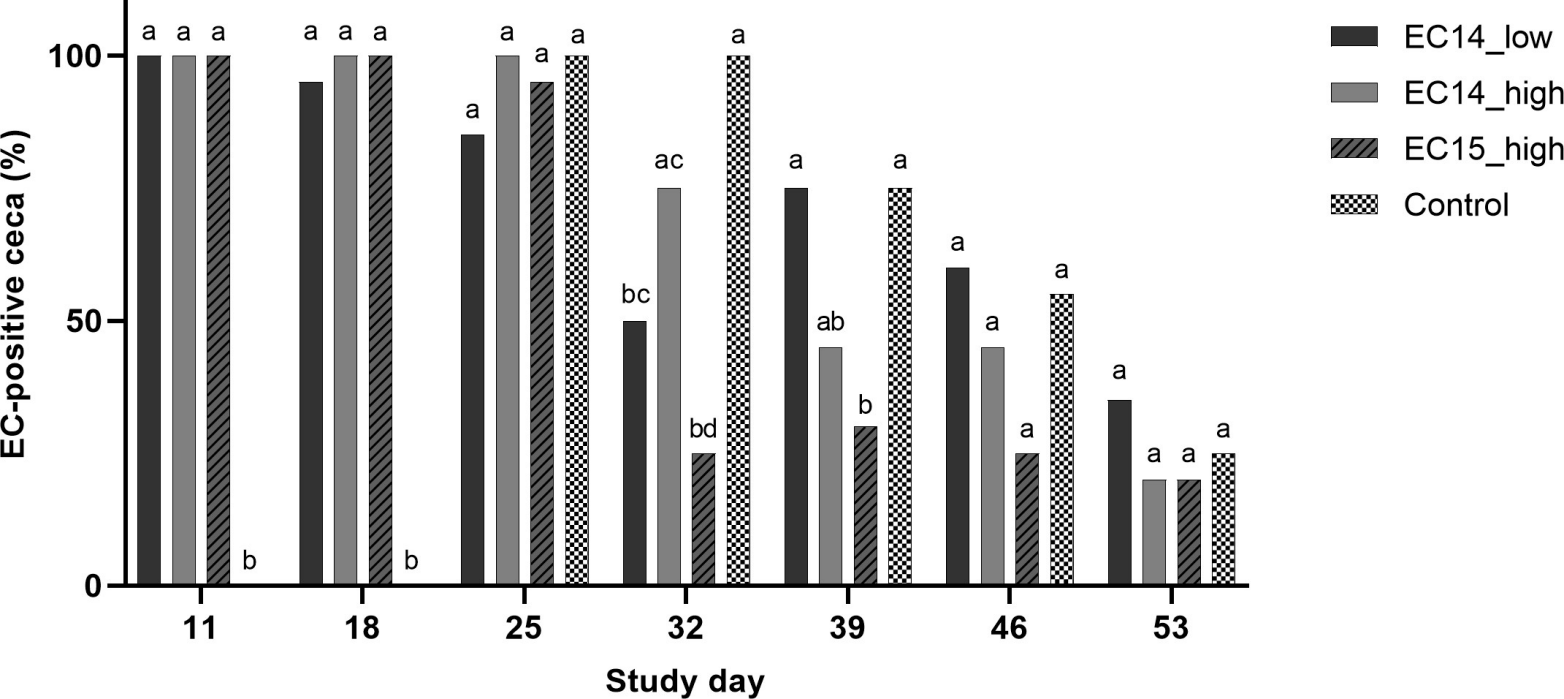
Cecal colonization by EC. Samples were analyzed via real-time PCR, and Ct values below 36 were considered positive. Different letters indicate significant differences between the groups per study day (*p* ≤ 0.05). Comparison between the groups was made for each study day by using Fisher’s exact test. *p*-value adjustments for multiple testing were performed by using the Bonferroni-Holm correction method. N = 20 per group and study day.

Cecal swabs were taken from all the birds at each necropsy day for real-time PCR in order to investigate the cecal colonization by EC throughout the trial. We included the EC15_high group in this analysis as we wanted to see, if this strain actually colonized the gut of the broilers without causing the EC-associated disease. EC was detected in nearly 100% of the birds in our infected groups (EC14_low, EC14_high and EC15_high) at study days 11 and 18. Most of the chickens in these groups were still EC-positive at study day 25. The control group was EC-negative at study days 11 and 18. At study days 25 and 32, 100% of the broilers in the control group were EC-positive in the cecum. Thereafter, the EC detection rate in the cecum then decreased until the end of the experiment in all four study groups (Fig 5).

## Discussion

*Enterococcus cecorum* is a serious threat to the broiler industry, causing high economic losses worldwide. To date, little is known about EC pathogenesis, and prevention of the associated disease is highly important. The overall aim of the present study was to establish a reliable infection model of the EC-associated disease. We successfully reproduced the EC-associated disease. This experimental reproduction was shown to be strain dependent, thus emphasizing the need to distinguish virulent and commensal strains. Oral transmission is the most likely infection route of EC [3], although other infection routes have been proposed as well [34]. Since pathogenic EC has been reported to colonize broiler chicks during the first week of life [10, 16], and experimental reproduction has been successful after oral inoculation at the first day of life [10], we decided to use similar parameters. The broiler chicks were inoculated at the first day of life using two different EC strains (EC14 and EC15) at two different concentrations each (10^6^ CFU/mL and 10^8^ CFU/mL). We aimed to compare the two strains in regard to their ability to cause the EC-associated disease. In the EC14 groups, EC was recovered from extraintestinal organs in approximately 23% of the birds via culture. This detection rate is in accordance with morbidity rates of up to 35% reported in the literature [9, 10, 15, 25]. In addition, in the two EC14 groups, birds showing severe symptoms of the EC-associated disease had to be euthanized due to animal welfare reasons. These EC-associated deaths led to higher mortality in the EC14 groups in comparison to the EC15 groups and the control group. The EC-associated mortality from our experiment can be linked to mortality rates from field outbreaks, which are elevated due to EC infection but generally do not exceed 15% [9, 10, 15, 24, 25]. Although clinical symptoms of the septic phase of the EC associated disease, such as depression and ruffled feathers, are rather unspecific [11], they were more often found in the EC14 groups than in the EC15 groups and the control group. This also applied for pericarditis and perihepatitis. Lameness, the main clinical symptom of the skeletal phase, was primarily found in both EC14 infected groups from the fourth week onwards until the end of the study. Additionally, typical bone lesions, such as spondylitis at the FTV and femoral head osteomyelitis, were only found in the EC14 groups. The bacteriological examination via culture showed that clinical symptoms and gross lesions observed in the EC15 groups and the control group could not be associated with EC and underlined their unspecific nature, since EC was not reisolated from affected birds in these groups. The EC detection rates via culture and real-time PCR in the spleen peaked at study day 18 in group EC14_low, thus representing the peak of the septic phase as reported for field cases [9] and experimental infection [10]. At study day 30, the first bird from group EC14_high was euthanized due to complete paralysis. Most positive FTVs were found between study days 32 and 46 in both EC14 groups. This is also in accordance with case reports on EC, where spondylitis mainly occurred from week three until the end of the production cycle [4, 7, 9, 14]. In the control group, 3% of the FTVs and 2% of the femoral heads were found to be EC-positive although no gross lesions were observed at the respective sampling site. Transmission from the EC-infected groups is rather unlikely, as each group was housed in a separate room without direct or indirect contact between infected and non-infected birds. It is more likely that contamination occured during the sampling procedure at necropsy or that false positive results occurred in real-time PCR. Based on the results from clinical observations, pathology, bacteriological examination, and real-time PCR, we can conclude that EC14 is a suitable strain to experimentally reproduce the EC-associated disease in broilers.

Interestingly, the EC15 strain did not cause any kind of clinical disease or pathologic changes associated with the EC infection, regardless of the inoculum concentration. EC15 was only isolated from the extraintestinal organs of two broilers in the EC15_high group and not at all in the EC15_low group on culture. We decided to analyze cecal swabs via real-time PCR to figure out if the birds in the EC15_high group were actually colonized by EC after oral inoculation. We could show that EC was present in the EC15_high group at study day 11 and throughout the whole trial. Cecal EC colonization was highly comparable between the EC14 groups and group EC15_high, whereas the control group was not colonized by EC before study day 25. EC colonization in the control group was in accordance with the literature, where it has been reported that commensal strains start to colonize the gut in the third week of life [10, 16]. Although our real-time PCR assay is specific for EC and highly sensitive, it cannot distinguish between different EC strains [16]. As we did not isolate and sequence the respective strain from the control birds, it remains unclear where this strain originated from and whether it was a pathogenic one or not. It seems to be most likely that the control group was colonized by a commensal EC strain. Furthermore, we suggest that EC15 colonized the gut in the EC15 groups after inoculation, but was not able to translocate from the intestine and actually cause the disease. As the inoculum concentration was calculated by determining the total bacterial count and EC-DNA was detectable in the gut of chickens in the EC15_high group throughout the entire experiment, it is unlikely that EC15 did not colonize birds in the EC15 groups. Prior to the experiment, we expected EC15 to cause the EC-associated disease, as the strain induced high embryo lethality in a respective assay [21]. Embryo lethality assays have been used frequently to distinguish between pathogenic and commensal strains [21, 26, 27]. Nevertheless, it has been recently discussed whether embryo lethality assays are reliable tools to determine EC pathogenicity. In a recent study, an EC strain isolated from severe bone lesions had low pathogenicity in an embryo lethality assay, leading to the conclusion that those assays are not the most representative measures to determine EC pathogenicity [28]. Our findings strengthen this hypothesis. In fact, high embryo lethality of EC15 was not accompanied by high virulence in our experiment. It is possible that EC15 is less virulent than EC14 and would have needed additional predisposing factors to translocate and cause the disease. However, our results represent the first proof that pathogenicity varies among EC strains in experimentally infected broilers. Further research on the genomic level is needed to characterize EC pathogenicity and cecal colonization, to find virulence factors, and to completely understand the pathogenic mechanisms of EC.

Besides the ability of the strains to cause the disease, we also aimed to find a suitable infectious dose for further animal experiments. We used an infectious dose of 10^6^ CFU in group EC14_low and 10^8^ CFU in group EC14_high. No clear differences could be detected concerning clinical signs and gross lesions between the two EC14 groups. Furthermore, we could not find any significant differences between the two EC14 groups in the bacteriological examination via culture and real-time PCR. In addition, the time course of the disease was only slightly different between the two EC14 groups. Infectious doses of 10^8^ CFU were used in two former studies to reproduce the EC-associated disease [3, 10]. In our experiment, we could show that a single oral inoculation with 10^6^ CFU of the pathogenic EC14 strain at the first day of life was perfectly sufficient. Moreover, we could confirm that no additional predisposing external factors were needed to reproduce the EC-associated disease in broiler-type chickens.

Interestingly, unspecific clinical signs and pathologic lesions of the heart and liver were seen in all groups in this experiment, but the amount of symptomatic birds was higher in the EC14 groups. The number of pathologic lesions and EC-positive culture results for all examined organs was slightly inconsistent in the EC14 groups. It is possible that EC was isolated from macroscopically still unchanged organs as the birds were randomly submitted to weekly necropsies. In addition, clinical signs as well as gross lesions reported here are rather unspecific and can be seen in several bacterial diseases in broilers including those caused by *Escherichia coli* or *Staphylococcus aureus* [11, 15]. This fact would also explain symptoms and gross lesions observed in the EC15 groups and the control group. As CNA agar, an agar selective for gram-positive cocci, was used for bacterial cultivation, we may have missed isolation of *Escherichia coli* from gross lesions in those groups.

Furthermore, it has been reported that birds can develop gross lesions without showing any clinical signs during the septic phase in field cases, and EC was isolated from the spleen of asymptomatic birds after natural and experimental infection [9, 10]. In addition to bacterial cultivation, we used real-time PCR to detect EC in different organs. Spleens from all the birds were examined using both methods. In general, results from the two different methods were highly comparable. We found a similar amount of EC-positive spleens via culture and real-time PCR, but at some time points, different individuals were found to be positive with the two methods. This could be due to the sampling procedure, as two different swabs were used for the two methods. It has been reported that EC can only be isolated with varying success from chronic cases [16]. Our real-time PCR was found to be highly sensitive and specific for EC [16], but the real-time PCR detects the presence of EC-DNA, whereas bacterial cultivation only detects viable EC. Using real-time PCR, EC-DNA was detected in predisposed articulations without any gross lesions found at necropsy. It is possible that the respective individuals were submitted to necropsy before macroscopic lesions could develop. Accordingly, we can underline that screening a flock throughout the production cycle and considering clinical observations, pathologic lesions, and bacteriological examination of gross lesions and predisposed articulations in close context to each other are essential in regard to EC infections. This enables farmers and veterinarians to detect ongoing EC outbreaks as soon as possible and to start treatment in time, as preventive methods such as vaccination are still rare [11].

Throughout the entire experiment, no significant differences in BW between the EC14 groups and the control group were found and birds in all three groups fulfilled the criteria as defined in the Performance Objectives by Aviagen [35]. When comparing EC-positive birds and EC-negative birds from the two EC14 groups, significantly lower BW were found in EC-positive birds at some time points. Similar results were found by Borst *et al*. in 2017 [10], who found enterococcal spondylitis to be significantly associated with decreased BW.

Overall, all these findings reported here further underline the facultative pathogenic nature of EC. Not all of the animals in a flock are affected by the disease in the field as was the case in our experiment, although all birds in the EC14 groups were colonized by EC after experimental infection for the first three weeks of the experiment. Different explanations have been given in the literature, and predisposing conditions, such as coinfection with other enteric pathogens and osteochondrosis dissecans, have been discussed and investigated [10, 18]. Intestinal integrity and the gut microbiota are also considered as potential factors influencing EC pathogenesis [18]. However, it remains unclear why some broilers in a flock are affected and others not. Further research is needed to find relevant host factors, virulence factors, and external factors, and to completely understand the pathogenesis of the EC-associated disease in individual birds.

In conclusion, we have successfully established an infection model for EC in broilers that represents the situation in the field. This experimental reproduction was shown to be strain-dependent and underlines the need to definitely distinguish virulent and commensal strains in further research on EC pathogenesis. We used different detection methods in our experiment to monitor the course of the disease and could show that our infection model reflects data from field outbreaks. Different diagnostic methods, such as bacteriological examination via culture and real-time PCR, can complement each other very well and can often detect EC in extraintestinal organs even before macroscopic lesions develop. This enables an early onset of intervention strategies in the field to avoid high economic losses. Further research on the EC pathogenesis and possible pre- and intervention strategies are still needed to combat EC in the broiler industry.

## Supporting information

S1 Fig Comparison of body weight between EC-positive and EC-negative birds. Data from the two EC14 groups were summarized and the EC-status based on total culture results was used as dependent variable. The Mann-Whitney *U* test was used to compare the body weight between EC-positive and EC-negative birds per study day. Differences were considered significant at *p* ≤ 0.05.
(TIF)

S2 Fig Comparison of EC-associated gross lesions and EC-positive real-time PCR results for FTV and femoral heads. (A) EC-positive free thoracic vertebrae (FTV) in % per study day. (B) EC-positive femoral heads (FH) in % per study day. Ct values below 36 were considered positive. N = 20 per group and day.
(TIF)
