# Supplementary figures and images for: Different virulence levels of Enterococcus cecorum strains in experimentally infected meat-type chickens
Source: PLoS One. 2021 Nov 12;16(11):e0259904. doi: 10.1371/journal.pone.0259904 (PMC8589174; doi:10.1371/journal.pone.0259904)

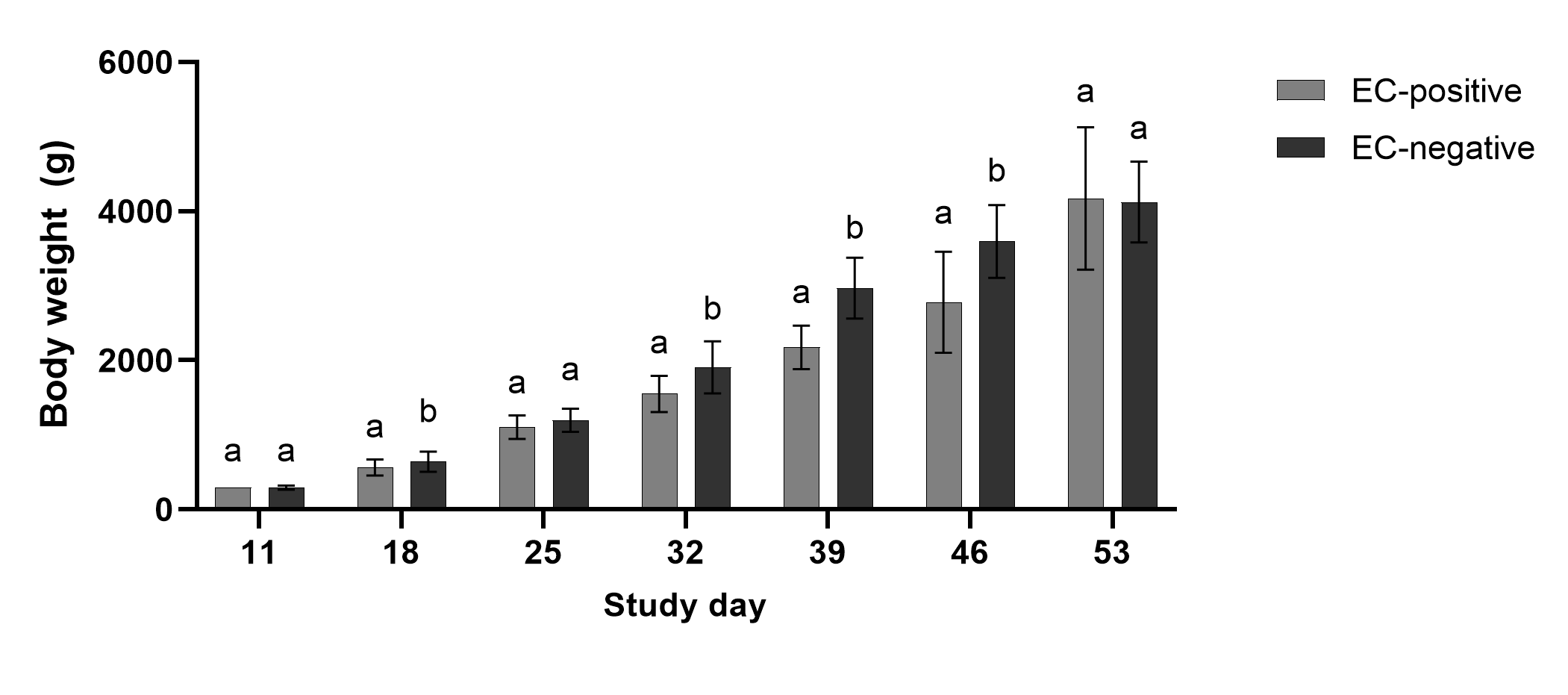

Supplement: S1 Fig — Data from the two EC14 groups were summarized and the EC-status based on total culture results was used as dependent variable. The Mann-Whitney U test was used to compare the body weight between EC-positive and EC-negative birds per study day. Differences were considered significant at p ≤ 0.05. (TIF) [file pone.0259904.s001.tif]

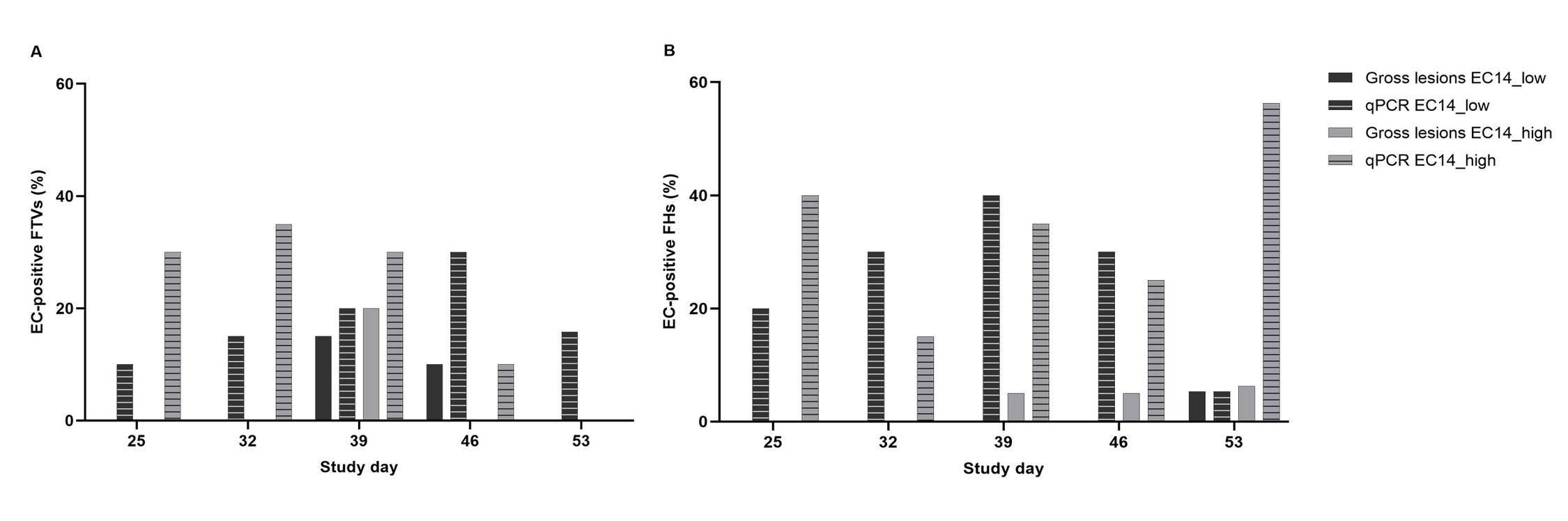

Supplement: S2 Fig — (A) EC-positive free thoracic vertebrae (FTV) in % per study day. (B) EC-positive femoral heads (FH) in % per study day. Ct values below 36 were considered positive. N = 20 per group and day. (TIF) [file pone.0259904.s002.tif]
